# Varied effectiveness of outpatient rehabilitation interventions in reducing long-term complications following childhood brain tumours: a systematic review

**DOI:** 10.3389/fresc.2026.1819083

**Published:** 2026-06-01

**Authors:** Jwalita Manikandan, Sophia Kennedy, Madhumita Dandapani, Jo Leonardi-Bee

**Affiliations:** 1 Children’s Brain Tumour Research Centre, Biodiscovery Institute, University of Nottingham, Nottingham, United Kingdom; 2 Centre for Evidence Based Healthcare, School of Medicine, University of Nottingham, Nottingham, United Kingdom

**Keywords:** brain tumour, cognitive, intervention, physical therapy, rehabilitation, systematic review

## Abstract

**Introduction:**

Brain tumours are a commonly diagnosed solid cancer in children and young adults. Many survivors experience long-term complications. We conducted a systematic review to assess the effectiveness of outpatient rehabilitation interventions in reducing long-term complications following childhood brain tumours.

**Materials and methods:**

Three databases were searched to November 2024. Two reviewers independently screened studies, assessed quality, and extracted data. Outcomes were categorised as primary [functional, psychological and Quality of Life (QoL)] and secondary (social, cognitive and sleep disturbance).

**Results:**

Eight papers were included. Most studies evaluated physical therapy interventions, assessing changes in functional outcomes, which were associated with the greatest proportion of significant improvements (*p* ≤ 0.05). Varying effects were seen for psychological, QoL, and social outcomes. Cognitive outcomes did not show significant improvements following cognitive, academic and exercise-based interventions.

**Discussion:**

Although studies reported positive functional, psychological, QoL and social outcomes, following various interventions, clear conclusions of the long-term effectiveness of rehabilitation interventions cannot be made. Further research is needed with longer-term follow-up, so effective rehabilitation strategies can be determined for this population. Better research could help develop interventions at a health-system level that could improve function, perceived wellbeing, and QoL of children and young people with brain tumours.

## Introduction

In the UK, it is estimated that more than 400 children are diagnosed with brain tumours every year (1). Because of developments in treatments and diagnoses, it is reported that the overall 5-year survival rate of childhood brain and central nervous system tumours is around 75%; however, survival remains highly variable, with outcomes varying markedly by tumour type, and benign and malignant tumours (2).

Studies suggest brain maturation is not complete until around 25 years of age, meaning childhood brain tumours are a developmental disorder (3, 4). This renders children particularly sensitive to brain tumours and their treatments (5). It is well known that brain tumour treatments, especially radiation can be damaging, leading to devastating long-term sequalae. These include but are not limited to physical, psychological, communication, endocrine and developmental disorders (6). Studies have identified that being of a younger age during diagnosis and treatment may increase the risk of long-term neurocognitive deficits (7), including deficits in working memory, executive function, attention and processing speed (8). These impairments leave many childhood brain tumour survivors unable to return to their daily activities before treatment, such as going to school or work, and many require lifelong care (9). Furthermore, the stress of brain tumour diagnoses, intensity of treatments as well as social isolation from not attending school, can lead to many patients becoming depressed (10). Other complications include cognitive problems associated with memory and attention (11). These long-term complications can greatly reduce the quality of life (QoL) of patients but also provide huge burdens to family members, who may have to leave work to become full-time carers, giving rise to further socioeconomic issues (12).

Rehabilitation interventions aim to reduce long-term complications and increase independence of brain tumour survivors in their activities of daily living (ADL) (13). Commonly used interventions include physiotherapy, occupational therapy, speech and language therapy and cognitive behavioural therapy (14). Rehabilitation can occur while the patient is still in hospital or after they have been discharged, as an outpatient. Interventions can differ in many ways; they can be interdisciplinary or multidisciplinary; they can aim to improve specific complications such as ataxia or hemiplegia, or improve patients overall QoL (15, 16). New rehabilitation interventions continue to be developed, and unconventional interventions such as video gaming have been investigated to improve physical and cognitive functioning sequalae in childhood brain tumour survivors (17, 18). Successful rehabilitation can have massive effects such as, contributing to patients' reintegration to society, improved physical and mental health and increased overall wellbeing (19).

To date, there has been no comprehensive review of the effectiveness of rehabilitation interventions following treatment to improve the long-term sequalae and decline in QoL experienced by many patients. Therefore, this systematic review aims to summarise evidence on the effectiveness of outpatient rehabilitation interventions in reducing long-term complications following brain tumours in children and young people (CYP) under 40 years.

## Materials and methods

This review adhered to the PRISMA and the Synthesis Without Meta-Analysis (SWiM) reporting guidelines (20, 21). The protocol for this review was registered with PROSPERO (CRD number redacted for anonymity).

### Inclusion and exclusion criteria

#### Population

People who had brain tumours during childhood aged up to 40 years were included. In instances where studies included participants over 40 years old, they were excluded if the mean age of participants was over 40 years old. Studies with less than 50% participants who had brain tumours were excluded.

#### Intervention

Any outpatient rehabilitation interventions were considered, including but not limited to, speech and language therapy, physiotherapy, occupational therapy and psychological therapy.

#### Comparison

Comparators included standard care, alternative rehabilitation interventions, baseline/pre-intervention values or no comparator.

### Outcomes

Primary outcomes included (i) Functional outcomes (balance, stability, coordination, motor skills, strength and ADL); (ii) Psychological outcomes (depression, anxiety, self-esteem, behavioural and emotional functioning); (iii) Quality of life (QoL).

Secondary outcomes included (i) Social outcomes (attendance at school and work, friendships, social re-integration, empathy and cooperation); (ii) Cognitive outcomes (processing speed, memory, attention, executive functioning, and mathematical fluency); (iii) Fatigue and sleep disturbances.

#### Study types

Randomised controlled trials (RCTs), quasi-experimental studies, cohort studies and case series were included. Systematic reviews, animal studies, case reports, and studies reported solely as abstracts were excluded.

### Searching, screening and data extraction

Three databases (MEDLINE, EMBASE, and PEDro) were searched from inception to November 2024 to identify eligible studies (Supplementary Table S1 for MEDLINE search). Hits from the searches were imported to EndNote (version 21) and then uploaded to Covidence (22), and duplicates were removed. Titles and abstracts were screened independently by two reviewers (JM and SK). Full text screening was then performed on the potentially eligible sources independently by two reviewers (JM and SK). Studies published in languages other than English were excluded at the full text stage due to translation not being sourced. Two reviewers (JM and SK) independently extracted data in Covidence (22). Any disagreements were resolved by consensus. Extracted data included (i) Study characteristics (first author details, year of publication, country, funding and conflicts of interest), (ii) Participant characteristics (mean age, sex, type of tumour and type of complication, total sample size, number of withdrawals, reason for withdrawals), (iii) Methodology (study design, group, blinding, randomisation and patient recruitment), (iv) Intervention (intervention, control, number of participants allocated, frequency, duration and setting), (v) Outcomes (outcome reporting, scale, range, unit of measurement, direction, data value, duration of follow up), and (vi) Results data

### Critical appraisal

Two reviewers (JM and SK) independently critically appraised the included studies using the JBI critical appraisal tool appropriate to the study design (23–25). Disagreements were resolved through discussion.

### Data synthesis

Due to the differences in study designs, interventions and outcomes measures of the included studies, it was not possible to conduct a meta-analysis. Therefore, results were synthesised using a narrative synthesis where studies were categorised by outcome measures. The standard metric used was two-sided *p*-values, and vote counting was used to compare the number of outcome measures had statistically significant *p*-values (*p* < 0.05). Subgroup analysis and an assessment of publication bias were not possible due to insufficient studies.

## Results

**Figure 1:**
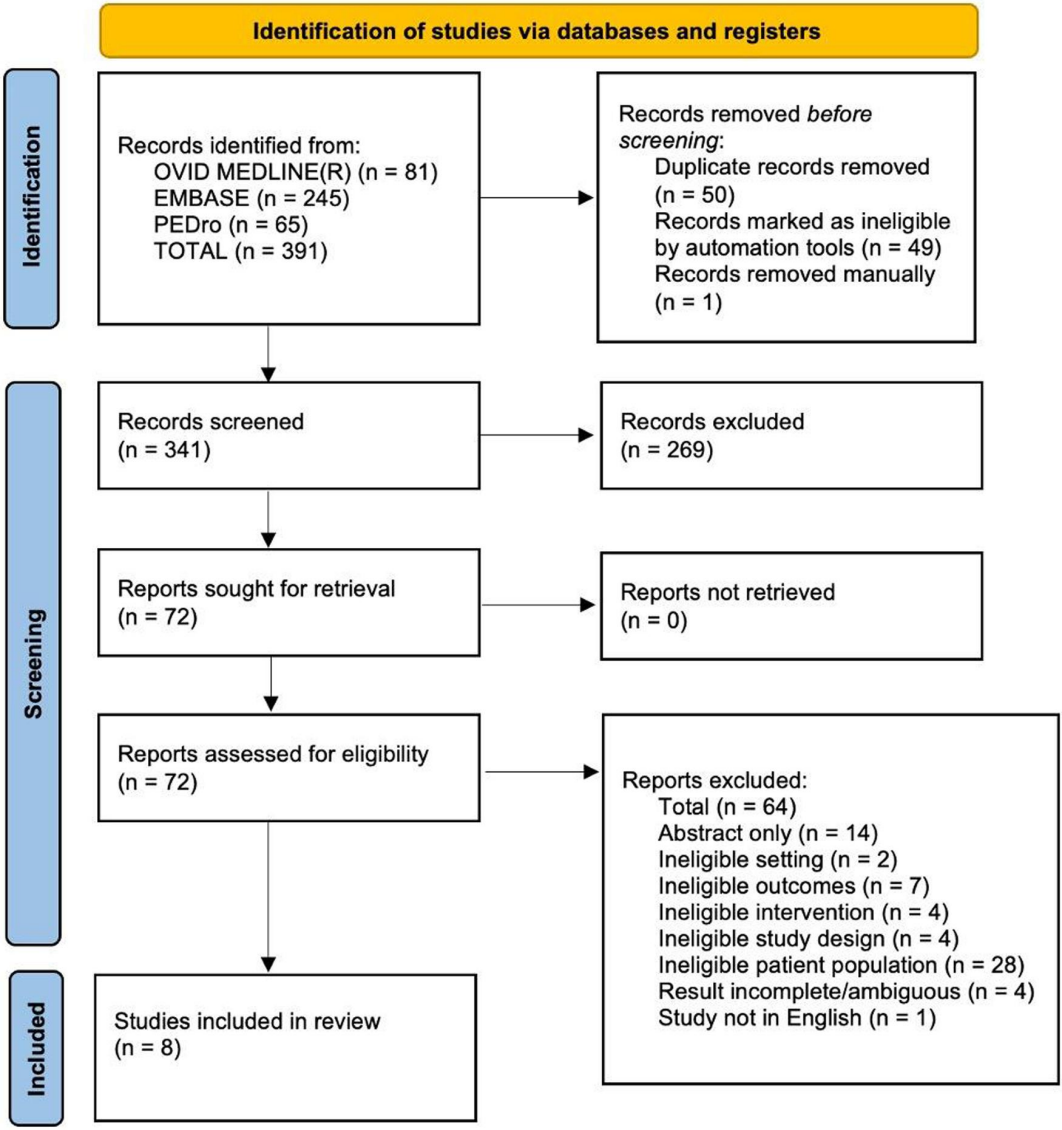
PRISMA flow chart.

The search identified 391 studies. Following de-duplication, 341 papers were screened at title and abstract stage, of which 72 studies were then carried forward to full-text review. Eight studies were included in the review (15–18, 26–29). Sixty-four studies were excluded predominately due to ineligible population (28 studies) or being published solely as an abstract (14 studies) (Figure 1).

The eight included studies had sample sizes ranging from 9 to 60 participants (Table 1). Most of the included studies used a RCT design; however, two further studies used a case-series design and a quasi-experimental design (16, 27). Four studies specified participants had a specific complication including upper-extremity hemiplegia, ataxia, depressive symptoms and cognitive deficits (15, 16, 26, 27). The studies were varied in terms of outcomes assessed and outcome measures used, with approximately 50 different scales utilised. Most studies investigated outcomes from physical therapy interventions (15–18, 29). Two studies investigated cognitive and academic interventions, and one investigated a musical training intervention (26–28).

**Table 1:** Characteristics of included studies.

Author, year	Country	Study design	Sample size (mean age), M/F	Intervention	Frequency/Duration	Outcomes (outcome scale)	Location of tumour
Cheung, 2018 (26)	China	RCT	60 (13.25 yrs) 35/25	One-on-one musical instrument training at home, tailored to their interests and abilities, provided by qualified musicians.	1 session per week/52 weeks	Depressive symptoms (CES-DC) Self-esteem (RSES) QoL (PedsQL 4.0)	NR
Patel, 2009 (27)	USA	Case series	12* (11.75 yrs) 6/6	Training program to teach compensatory learning and problem-solving skills in survivors with cognitive deficits.	1 session per week/3 to 6 months	Behavioural and emotional functioning (CBCL) Social behaviours (SSRS) Working Memory (WISC-III) Mathematical Fluency (WRAT3) Attention (43)	NR
Peterson, 2022 (28)	Canada	RCT, pilot study, parallel group (1:1:1)	29 (11.52 yrs) 21/7	Cogmed - a computer based, game-like exercises targeting visual-spatial working memory skills OR JumpMath- workbooks in line with local school curriculum meant to be completed with parents.	5 days per week/10 to 12 weeks	Working memory (WISC-IV and WRAML-2) Mathematic fluency (WJ-III)	53.6% Posterior fossa 28.6% Cerebral hemispheres 10.7% Suprasellar 7.1% Pineoblastoma
Sabel, 2016 (17)	Sweden	RCT, pilot study, crossover group	13 (12.5 yrs) 6/7	Active video-gaming using off-the-shelf, motion-controlled video console.	5 days per week/10 to 12 weeks	Body coordination (BOT-2) Manual coordination (BOT-2) Strength and Agility (BOT-2) · Fine manual control (BOT-2)	23.1% Posterior fossa 76.9% Supratentorial
Sabel, 2017 (18)	Sweden	RCT, pilot study, crossover groups	13 (12.5 yrs) 6/7	Off-the-shelf, motion-controlled video console, was used for home-based physical exercise (active video-gaming).	5 days per week/10 to 12 weeks	Motor and process skills (AMPS score) Cognitive outcomes (28 cognitive tests used)	23% Posterior fossa 77% Supratentorial
Selim, 2023 (15)	Egypt	RCT	30 (6.87 yrs) 15/15	Physical therapy programme including exercises for mobility, balance and gait training as well as a dual-task program (balance and cognitive).	3 sessions per week/8 weeks	Balance (PBS) Stability (standing stability IQR) Functional Independence (WeeFIM)	100% Posterior fossa
Sparrow, 2016 (16)	USA	Quasi-experimental study, Pilot study	9 (7.30 yrs)3/6	Participants with upper-extremity hemiplegia undergone constraint-induced movement therapy sessions.	5 days per week/3 weeks	Quality and frequency of use of affected arm (PAFT) Motor skills of non-functional arm (PMAL) Health related QoL (PedsQL SF-15 and Acute version) Activities of daily living (INMAP)	11.1% Suprasellar thalamus 11.1% Right hemisphere 11.1% Temporal lobe 11.1% Left frontoparietal 11.1% Frontoparietal 22.2% Thalamus 11.1% Midbrain and thalamus 11.1% Medullary
Usama, 2023 (29)	Egypt	RCT, phase III, parallel group (1:1:1)	60 (7.18 yrs) NR	HUMAC balance and tilt program OR coordination exercises along with Pilates core stability exercises.	3 sessions per week/3 months	Stability (LOS and COP) Bilateral coordination (BOT-2) Upper limb coordination (BOT-2) Balance (mCTSIB)	100% Posterior fossa

LOS, Limit of stability; COP, Centre of pressure; BOT-2, Bruininks-Oseretsky Test of Motor Proficiency, Second Edition; mCTSIB, Modified Clinical Test of Sensory Integration of Balance; PAFT, Paediatric Arm Function Test; PMAL, Paediatric Motor Activity Log; INMAP, Inventory of New Motor Activities and Programs; AMPS, Assessment of Motor and Process Skills; WISC-IV, Wechsler Intelligence Scale for Children-IV; WRAML-2, Wide Range Assessment of Memory and Learning Second Edition; WJ-III, Woodcock-Johnson III Tests of Achievement; PBS, Paediatric Balance Score; WeeFIM, Functional Independence Measure for Children; CES-DC, Center for Epidemiological Studies Depression Scale for Children; RSES, Rosenberg Self-Esteem Scale; PedsQL, Pediatric Quality of Life Inventory; CBCL, Child Behavior Checklist; SSRS, Social Skills Rating System; WISC-III, Wechsler Intelligence Scale for Children-III; WRAT3, The Wide Range Achievement Test 3; CPT, Conners’ Continuous Performance Test. Yrs, years. NR Not Reported. * 9 out of 12 participants had brain tumour.

### Critical appraisal

The scores for the studies ranged from 8.5 to 10 out of a maximum of 13 (Supplementary Table S2). Studies tended to score poorly and be at a risk of bias relating to allocation concealment, blinding of intervention to participants and those delivering the intervention. In contrast, all studies used true randomisation, with some studies using methods to consider potential confounders and balance treatment groups baseline characteristics, such as the minimisation method and stratified randomisation (15, 17, 18). However, two studies had differences in baseline characteristics between the intervention and control groups (28, 29).

The quasi-experimental study used an uncontrolled before-after design and hence scored poorly for risk of bias primarily due to not having a comparator group (16) (Supplementary Table S3). In contrast the case series study scored higher (8/10) (27) but was at a high risk of bias relating to the cases not being consecutive and incomplete inclusion of participants (Supplementary Table S3).

### Effectiveness of the interventions

#### Functional outcomes

Of the included studies, five measured the effectiveness of physical therapy interventions using functional outcomes including changes in balance, stability, coordination, strength, motor skills and functional independence/execution of ADL (15–18, 29) (Table 2). Most of these studies utilised a physical therapy intervention. Changes in functional outcomes were the most consistent improvements reported, with four studies reporting significant improvements in at least one functional outcome (15, 17, 18, 29). In Selim et al. reported significant effects in improving stability and balance (15). The postural stability group and the coordination group from Usama et al. had statistically significant improvements in upper limb and bilateral coordination (29), whereas the active video-gaming intervention from Sabel et al., 2016 showed no significant improvement in the body coordination (17). Sabel et al., 2016 and Sparrow et al. both measured changes in motor skills (16, 17). While active video-gaming did not lead to significant improvements in manual control, Sparrow et al. reported significant improvement in participants motor skills, following constraint-induced movement therapy (CIMT) (16). Of the three studies reporting ADL outcomes, significant improvements were only reported by Sparrow et al. in brain tumour survivors with upper-extremity hemiplegia (16).

**Table 2:** Effectiveness of outpatient rehabilitation interventions on functional outcomes.

Functional Outcomes	Sabel, 2016* (17)	Sabel, 2017* (18)	Selim, 2023 (15)	Sparrow, 2016 (16)	Usama, 2023 (29)	
Intervention	Active video gaming	Active video gaming	Dual-task programme	Constraint-induced movement therapy	Postural Stability	Coordination
Stability	NR	NR	*p* = 0.0001	NR	*p* < 0.0001	*p* = 0.15
					*p* < 0.0001	*p* = 0.56
Coordination	*p* = 0.37	NR	NR	NR	*p* = 0.0012	*p* < 0.0001
	*p* = 0.28				*p* < 0.005	*p* < 0.0001
Balance	NR	NR	*p* = 0.028	NR	*p* < 0.0001	*p* = 0.019
					*p* = 0.0002	*p* = 0.92
					*p* < 0.0001	*p* = 0.43
					*p* < 0.0001	*p* = 0.16
Gross and fine motor skills	*p* = 0.10	NR	NR	*p* < 0.001	NR	NR
				*p* < 0.001		
				*p* < 0.001		
				*p* < 0.001		
Strength	*p* = 0.42	NR	NR	NR	NR	NR
Activities of daily living	NR	*p* = 0.059	*p* = 0.158	*p* < 0.001	NR	NR
		*p* = 0.0296		*p* < 0.001		

Shaded green=statistically significant (*p* ≤ 0.05); shaded red=non significant (*p* > 0.05); NR = outcome not reported; * = crossover trial, after first period values adjusted with ANCOVA was recorded.

#### Psychological outcomes

Two studies measured changes in psychological outcomes post-intervention with mixed results (26, 27). Cheung et al., 2018 showed significant benefits in two psychological outcomes (depressive symptoms and self-esteem) following a musical training intervention (*p* < 0.001 for both outcomes) (26). In contrast, Patel et al. 2009 assessing behavioural and emotional functioning, reported no significant effects following a cognitive and problem-solving intervention (*p* > 0.05) (Table 3) (27).

**Table 3:** Effectiveness of outpatient rehabilitation interventions on psychological outcomes.

Psychological Outcomes	Cheung, 2018 (26)	Patel, 2009 (27)
Intervention	Musical training	Cognitive and problem-solving training
Depressive Symptoms	*p* < 0.001	NR
Self-esteem	*p* < 0.001	NR
Behavioural and Emotional Functioning	NR	*p* = 0.25
		*p* = 0.08
		*p* = 0.18

Shaded green=statistically significant (*p* ≤ 0.05); shaded red=non significant (*p* > 0.05); NR = outcome not reported.

#### Quality of life (QoL) outcomes

QoL was not frequently assessed in the studies, with only two studies reporting QoL (16, 26). The musical training intervention showed a benefit in patients QoL (*p* < 0.001), but no significant improvement was reported after the CIMT (*p* = 0.08) (16, 26).

#### Social outcomes

There is limited evidence on the effectiveness of rehabilitation interventions in improving social outcomes in CYP brain tumour survivors. One study reported a significant positive increase in the mean post-intervention scores compared to the mean pre-intervention scores was reported for social behaviours (*p* = 0.04) (27).

#### Cognitive outcomes

Cognitive outcomes were reported in three studies (18, 27, 28). Cognitive outcomes including working memory, mathematical fluency and attention were assessed, with results consistently showing no significant improvements. Sabel et al., 2017 assessed various cognitive outcomes using 28 different cognitive measures (18). None of the tests showed significant improvement following the active video-gaming intervention, compared to the control group and only *p*-values for the cognitive tests relating to working memory and attention are included in Table 4. Most subtests assessing working memory and mathematical fluency did not show significant improvements post-intervention. No significant benefit was reported in attention following any intervention.

**Table 4:** Effectiveness of outpatient rehabilitation interventions on cognitive outcomes .

Cognitive Outcomes	Patel, 2009 (27)	Peterson, 2022 (28)		Sabel, 2017 (18)
Intervention	Cognitive and problem-solving training	Cogmed	JumpMath	Active video gaming
Working Memory	*p* = 0.15	*p* = 0.01	*p* = 0.01	*p* = 0.86
		*p* = 0.03	NS	*p* = 0.34
		NS	NS	*p* = 0.18
		NS	NS	
Mathematical Fluency	*p* = 0.18	NS	*p* = 0.02	NR
		NS	NS	
		NS	NS	
		NS	NS	
Attention	*p* = 0.22	NR	NR	*p* = 0.09
	*p* = 0.09			*p* = 0.078
	*p* = 0.28			*p* = 0.70
	*p* = 0.09			
	*p* = 0.94			

Shaded green=statistically significant (*p* ≤ 0.05); shaded red=non significant (*p* > 0.05); NR = outcome not reported; NS = not significant (study did not specify *p*-value, only that the result was not significant).

## Discussion

This systematic review assessed the effectiveness of outpatient rehabilitation interventions for childhood brain tumour survivors across five outcome domains: functional, psychological, QoL, social and cognitive outcomes. Most of the eight included studies utilised a physical therapy intervention and assessed changes in functional outcomes, where the greatest number of significant improvements were found (15, 17–19, 29). Positive trends were found associated with musical training on psychological outcomes; however, the findings for cognitive and problem-solving training were more mixed (26, 27). Mixed findings were seen for QoL (16, 26). Limited social outcomes were reported; however, a significant improvement in participants' perceived importance of social behaviours, following a problem-solving intervention (27) was seen. Of the cognitive outcomes assessed, generally no significant improvement in results were seen. None of the included studies reported fatigue and sleep disturbance.

In general, studies investigating physical therapy interventions such as the coordination and postural stability groups in Usama et al., the dual-task group in Selim et al. and the CIMT intervention in Sparrow et al., showed the greatest number of significant improvements of functional outcomes such as balance, stability, coordination, motor skills and ADL. A previous systematic review on the effect of exercise-based rehabilitation in paediatric brain tumour patients concluded that patients showed improvements in motor skills and overall physical fitness (30), which is consistent with our findings. They attributed these improvements to an increase in white matter in several parts of the brain including the pre- and post-central gyri, and parts of the left temporal lobe, following physical activity (30). Studies have indicated that physical activity and gross motor skills are related to an increase in white matter microstructure in children, which may be associated with greater neurocognitive function (31). This could suggest that physical therapy rehabilitation may not only improve functional outcomes, but also cognitive function.

The musical training intervention evaluated by Cheung et al., showed great potential in improving psychological and QoL outcomes, in those exhibiting depressive symptoms following brain tumours and their treatments (26). It was one of the only interventions where participants showed statistically significant improvements across all assessments. The intervention also had the longest duration of 52 weeks and the largest sample size of 60 compared to the other studies. This long duration may explain why the greatest effects were observed following this study. Duration of the interventions were hugely varied, ranging from 3 weeks to a year. The two studies measuring QoL in participants used different versions of PedsQL scale (16, 26). Sparrow et al. used a parent-reported version which did not yield significant improvement and reported that in participants individual results, two participants QoL decreased by the end of the intervention. Some parents reported that the CIMT intervention was frustrating, which could explain why not much improvement was reported in the participants overall QoL (16). In comparison to Sparrow et al. using a parent-reported PedsQL scale, Cheung et al. utilised a patient-reported PedsQL scale. From this, significant improvements in participants QoL were observed. Although these two interventions are massively varied, patient perception of their QoL may differ to that of their parents, which could have contributed to these disparate results.

Cheung et al. was the only study to report significant improvements in psychological outcomes (self-esteem and depressive symptoms), following the musical training intervention (26). Patel et al. investigating a cognitive and problem-solving intervention in those with cognitive deficits also assessed a psychological outcome—participants' behavioural and emotional functioning, with no significant benefits reported, compared to pre-intervention assessments (27). The effects of music in depression have been widely researched in multiple patient demographics such as older adults and those with dementia (32, 33). A previous systematic review investigating the effect of music therapy on people of all ages with depression found effectiveness in short term reduction of depressive symptoms and anxiety (34). Music therapies with greater time per session might be associated with stronger therapeutic effects on depression (35). Cheung et al. used 45-minute, weekly sessions and reported consistent improvements in depressive symptoms and self-esteem during the 6-month follow-up as well as the 12-month follow-up (26). However, no information was provided on whether effects were maintained after the intervention, or if they were transient.

In contrast to the functional outcomes, no statistically significant improvements in cognitive outcomes were seen following the interventions. Sciancalepore et al. conducted a systematic review on computer-based cognitive training interventions on children with brain tumours, finding temporary neurocognitive improvements that were only present when the interventions were used (36). These cognitive-based interventions had the greatest withdrawals and lowest recruitment rates in comparison to other interventions even though they were offered to participants for free. This suggests cognitive and academic interventions may be less appealing for patients and their families.

Furthermore, interventions that involve regular travel and long hours may not be sustainable in long-term, which could explain why interventions such as active video-gaming may be a more acceptable way for patients to rehabilitate with the convenience of doing it in their own time, at home (17, 18). However, a decrease in physical activity needs to be considered due to participants using video-gaming as an alternative to other exercise (18).

This systematic review has some limitations. Nine different interventions were assessed with the included studies reporting over 50 different outcome measures. This variability in the interventions and outcome measures made it difficult to directly compare results across studies. Therefore, we were unable to conduct a meta-analysis and synthesised the findings using vote counting based on *p*-values, hence, the likely magnitude of the intervention effect could not be quantified. Whilst we conducted a comprehensive search of electronic databases, we excluded grey literature, studies reported solely as abstracts, and those reported in languages other than English, which increases the possibility that some evidence was excluded from the review. All the included studies were limited by a small sample size, which may explain why non-significant *p*-values were seen in many studies due to a lack of power in the statistical analyses. However, research in people under 18 years is challenging as brain tumours in children are relatively rare, and there are many ethical and legal considerations (37). In addition, the varied location of these tumours combined with the range of developmental stages of the growing children means that often interventions are individualised, making comparisons difficult. We were unable to segregate the findings based on the modality of therapy due to insufficient reporting within the included studies. Although the investigated interventions would not allow participants to be blinded, this may influence their behaviour in the studies and impact on patient-reported outcomes (38).

Because of the limitations to this review as well as the included studies, it is difficult to provide evidence-based guidance for clinical practice. However, recommendations on further research can be made. Firstly, larger RCTs, with blinded assessors are needed for trials to have a large sufficient power to draw clinically significant conclusions (39, 40), where large cooperative international trials are needed due to the rarity of individual brain tumours. However, there remains additional challenges with incorporating standardised measures of neurocognition, mobility scores, and patient reported outcome measures (PROMs) that are pertinent to the diverse patient demographic as well as the individual treatment modalities for each tumour type. The duration of the interventions and the length of follow-up should be optimised to enable the determination of long-term effectiveness. Ideally, these interventions need to be assessed directly through head-to-head comparisons; however, sub cohorts based on the therapeutic option would need to be used to overcome the potential disparities in outcomes due to the modality of intervention, for example, when assessing surgery vs. multimodal therapy. Furthermore, the use of standardised and validated outcome measures in the brain tumour rehabilitation research field would improve the comparability of studies in the future. We found few interventions assessing social, psychological and QoL outcomes in comparison to functional and cognitive outcomes. This is surprising as these are well-known and commonly reported complications following brain tumours and their treatments (41, 42). Changes in sleep disturbance and fatigue outcomes were not assessed in any of the included studies which could be an area for further research to address. Patients' reintegration into society and school is also an important aspect of rehabilitation, therefore future studies including assessment of these outcomes could provide beneficial information on the effectiveness of an intervention.

## Conclusion

Several rehabilitation interventions included in this review showed promise in improving outcomes of various domains in patients following brain tumours; however, due to the limitations in the evidence, no firm conclusions can be made regarding which type of interventions are more effective or their long-term benefits. Further high-quality experimental research is needed with larger sample sizes. There is a need for more consistent, standardised outcome measures to be used across studies to support the synthesis of future evidence.

## Data Availability

The original contributions presented in the study are included in the article/Supplementary Material, further inquiries can be directed to the corresponding author.
