# Supplementary material for: Varied effectiveness of outpatient rehabilitation interventions in reducing long-term complications following childhood brain tumours: a systematic review
Source: Front Rehabil Sci. 2026 Jun 1;7:1819083. doi: 10.3389/fresc.2026.1819083 (PMC13265511; doi:10.3389/fresc.2026.1819083)
Supplement: Supplementary file 1 [file Datasheet1.docx]

#### Supplementary table 1: Medline search strategy

Ovid MEDLINE(R) ALL <1946 to November 07, 2024>

1 Brain Neoplasm*.mp.

2 Brain Neoplasms/

3 Central Nervous System Neoplasms/

4 Central Nervous System Neoplasm*.mp.

5 CNS Neoplasm*.mp.

6 Brain Cancer.mp.

7 Brain Tumo$r.mp.

8 Central Nervous System Tumo$rs.mp.

9 1 or 2 or 3 or 4 or 5 or 6 or 7 or 8

10 Rehabilitation/

11 Rehab*.mp.

12 Recover*.mp.

13 Intervention*.mp.

14 10 or 11 or 12 or 13

15 Outpatients/

16 Outpatient*.mp.

17 Ambulatory Care/

18 Ambulatory.mp.

19 Home Care Services/

20 15 or 16 or 17 or 18 or 19

21 9 and 14 and 20

22 limit 21 to ("all infant (birth to 23 months)" or "all child (0 to 18 years)" or "all adult (19 plus years)" or "newborn infant (birth to 1 month)" or "infant (1 to 23 months)" or "preschool child (2 to 5 years)" or "child (6 to 12 years)" or "adolescent (13 to 18 years)" or "young adult (19 to 24 years)" or "adult (19 to 44 years)" or "young adult and adult (19-24 and 19-44)")

Supplementary table 2: JBI Critical Appraisal tool for Randomised Controlled Trials

| **Question** | **Cheung, 2018 [27]** | **Peterson, 2022 [26]** | **Sabel, 2016 [16]** | **Sabel, 2017 [17]** | **Selim, 2023 [14]** | **Usama, 2023 [25]** |
| --- | --- | --- | --- | --- | --- | --- |
| 1. Was true randomization used for assignment of participants to treatment groups? | **Y** | **Y** | **Y** | **Y** | **Y** | **Y** |
| 2. Was allocation to treatment groups concealed? | **Y** | **U** | **N** | **N** | **N** | **N** |
| 3. Were treatment groups similar at the baseline? | **Y** | **N** | **Y** | **Y** | **Y** | **N** |
| 4. Were participants blind to treatment assignment? | **N** | **N** | **N** | **N** | **N** | **N** |
| 5. Were those delivering the treatment blind to treatment assignment? | **N** | **N** | **N** | **N** | **N** | **U** |
| 6. Were treatment groups treated identically other than the intervention of interest? | **Y** | **Y** | **Y** | **Y** | **U** | **Y** |
| 7. Were outcome assessors blind to treatment assignment? | **N** | **Y** | **Y** | **Y** | **N** | **U** |
| 8. Were outcomes measured in the same way for treatment groups? | **Y** | **Y** | **Y** | **Y** | **Y** | **Y** |
| 9. Were outcomes measured in a reliable way | **Y** | **Y** | **Y** | **Y** | **Y** | **Y** |
| 10. Was follow up complete and if not, were differences between groups in terms of their follow up adequately described and analysed? | **Y** | **Y** | **Y** | **Y** | **Y** | **Y** |
| 11. Were participants analysed in the groups to which they were randomized? | **Y** | **Y** | **Y** | **U** | **Y** | **Y** |
| 12. Was appropriate statistical analysis used? | **Y** | **Y** | **Y** | **Y** | **Y** | **Y** |
| 13. Was the trial design appropriate and any deviations from the standard RCT design accounted for in the conduct and analysis of the trial? | **Y** | **Y** | **Y** | **Y** | **Y** | **Y** |
| Total score | **10** | **9.5** | **10** | **9.5** | **8.5** | **9** |

Y Yes, N No, U Unclear

Supplementary table 3: JBI Critical Appraisal tool for Quasi-experimental and Case series studies

| **Quasi-experimental questions** | **Sparrow, 2023 [15]** | **Case series questions** | **Patel, 2009 [28]** |
| --- | --- | --- | --- |
| 1. Is it clear in the study what is the “cause” and what is the “effect” | **Y** | 1. Were there clear criteria for inclusion in the case series? | **Y** |
| 2. Was there a control group? | **N** | 2. Was the condition measured in a standard, reliable way for all participants included in the case series? | **Y** |
| 3. Were participants included in any comparisons similar? | **-** | 3. Were valid methods used for identification of the condition for all participants included in the case series? | **Y** |
| 4. Were the participants included in any comparisons receiving similar treatment/care, other than the exposure or intervention of interest? | **-** | 4. Did the case series have consecutive inclusion of participants? | **N** |
| 5. Were there multiple measurements of the outcome, both pre and post the intervention/exposure? | **Y** | 5. Did the case series have complete inclusion of participants? | **N** |
| 6. Were the outcomes of participants included in any comparisons measured in the same way? | **-** | 6. Was there clear reporting of the demographics of the participants in the study? | **Y** |
| 7. Were outcomes measured in a reliable way? | **Y** | 7. Was there clear reporting of clinical information of the participants? | **Y** |
| 8. Was follow-up complete and if not, were differences between groups in terms of their follow-up adequately described and analysed? | **Y** | 8. Were the outcomes or follow up results of cases clearly reported? | **Y** |
| 9. Was appropriate statistical analysis used? | **Y** | 9. Was there clear reporting of the presenting site(s)/clinic(s) demographic information? | **Y** |
|  |  | 10. Was statistical analysis appropriate? | **Y** |
| Total score | **5** |  | **8** |

Y Yes, N No, U Unclear
